# Young and Older Adults Benefit From Sleep, but Not From Active Wakefulness for Memory Consolidation of What-Where-When Naturalistic Events

**DOI:** 10.3389/fnagi.2019.00058

**Published:** 2019-03-20

**Authors:** Kouloud Abichou, Valentina La Corte, Nicolas Hubert, Eric Orriols, Alexandre Gaston-Bellegarde, Serge Nicolas, Pascale Piolino

**Affiliations:** ^1^Laboratoire Mémoire Cerveau et Cognition (MC2Lab EA 7536), Institut de Psychologie, Université Sorbonne Paris Cité, Boulogne-Billancourt, France; ^2^Institute of Memory and Alzheimer’s Disease, Department of Neurology, Pitié-Salpêtrière Hospital, Paris, France; ^3^Institut Universitaire de France, Paris, France

**Keywords:** episodic memory, binding, What-Where-When task, consolidation, sleep, awake active state, aging, virtual reality

## Abstract

An extensive psychological literature shows that sleep actively promotes human episodic memory (EM) consolidation in younger adults. However, evidence for the benefit of sleep for EM consolidation in aging is still elusive. In addition, most of the previous studies used EM assessments that are very different from everyday life conditions and are far from considering all the hallmarks of this memory system. In this study, the effect of an extended period of sleep was compared to the effect of an extended period of active wakefulness on the EM consolidation of naturalistic events, using a novel (What-Where-When) EM task, rich in perceptual details and spatio-temporal context, presented in a virtual environment. We investigated the long-term What-Where-When and Details binding performances of young and elderly people before and after an interval of sleep or active wakefulness. Although we found a noticeable age-related decline in EM, both age groups benefited from sleep, but not from active wakefulness. In younger adults, only the period of sleep significantly enhanced the capacity to associate different components of EM (binding performance) and more specifically the free recall of what-when information. Interestingly, in the elderly, sleep significantly enhanced not only the recall of factual elements but also associated details and contextual information as well as the amount of high feature binding (i.e., What-Where-When and Details). Thus, this study evidences the benefit of sleep, and the detrimental effect of active wakefulness, on long-term feature binding, which is one of the core characteristics of EM, and its effectiveness in normal aging. However, further research should investigate whether this benefit is specific to sleep or more generally results from the effect of a post-learning period of reduced interference, which could also concern quiet wakefulness.

## Introduction

Episodic memory (EM) refers to personally experienced events, located in time and space, that are unique and whose retrieval depends on mentally traveling back in time to re-experience the previous encoding context (Tulving, 2002). Recalling episodic events requires binding factual and spatio-temporal (What-Where-When) information and generates a subjective awareness, termed autonoetic consciousness, that are important defining features of EM (Tulving, 1985). The notion of EM is also dynamic since it undergoes significant transformations from the time of encoding throughout the lifetime (Bartlett, 1932; Moscovitch et al., 2005; Piolino et al., 2009; Stickgold and Walker, 2013). In fact, the newly acquired memory is labile, and its transformation into a long-lasting memory trace is sustained by a dynamic process commonly referred to as memory consolidation.

According to the **Active Hypothesis,** also referred to as the **Unique-to-Sleep Consolidation Hypothesis**, sleep is a privileged state for memory consolidation (Walker and Stickgold, 2004; Ellenbogen et al., 2006a,b; Rasch et al., 2007; Payne et al., 2008; Diekelmann and Born, 2010; Rasch and Born, 2013). In younger adults, various behavioral studies have investigated the role of sleep stages on EM consolidation, both the rapid eye movement (REM) stage and the non-REM sleep (NREM) stage which is particularly characterized by slow wave sleep (SWS) (Diekelmann and Born, 2010). The active role of sleep in EM consolidation is assumed to be mediated by a dialogue between the hippocampus and the neocortex. During SWS (Buzsáki, 1998), the newly acquired traces are replayed in the hippocampus and are gradually transferred to cortical areas where they enter pre-existing networks. This transfer enables the stabilization and strengthening of new memory traces (Squire and Alvarez, 1995; McGaugh, 2000; Dudai, 2004; Diekelmann et al., 2009; Stickgold and Walker, 2013).

Although there is ample evidence to support the specific implication of sleep stages, particularly SWS, in memory consolidation, other findings (Hasselmo and McClelland, 1999; Mednick et al., 2011; Wixted and Cai, 2013) support the idea that while consolidation does occur during sleep, it is not unique to sleep. Some authors (Hasselmo and McClelland, 1999) suggest that memory consolidation benefits essentially from a state of reduced sensory input, including sleep (specifically SWS) but not limited to it (e.g., quiet wake). Accordingly, specific brain rhythmic activity (hippocampus high frequency sharp wave and ripples and neocortex low frequency spindle oscillations), as well as a decrease in acetylcholine (Ach) levels characterize the state of reduced sensory input in general and mediate the shift of the hippocampus from the encoding state to the consolidating state during which cortex representations are strengthened. In the same vein, the state of reduced cognitive activity has also been proposed to account for consolidation processes. According to the **Opportunistic hypothesis** (Mednick et al., 2011), a period of reduced learning and hippocampal activity (e.g., sleep and quiet wake) as opposed to a period in which the hippocampus is engaged in encoding new memories (e.g., active wake), favors consolidation by protecting acquired memories from interference.

Both theories support the involvement of sleep in memory consolidation, either through stabilization (opportunistic hypothesis) or enhancement (the active hypothesis). Moreover, both consider active wake as conducive to new encoding and unfavorable for consolidation.

It should be noted that, with increasing age, there are substantial changes in the quantity and quality of sleep, such as reduced sleep duration, reduced SWS and REM sleep, changes in sleep timing and spindle density but also changes in sleep continuity/fragmentation (Bliwise et al., 2009; Scullin and Bliwise, 2015). Moreover, given that EM is highly age-sensitive, an alluring question is whether older adults still benefit from sleep for memory consolidation as younger adults do.

Intriguingly, studies that examine the effect of aging on declarative memory consolidation have provided conflicting evidence. Some of them have reported that the benefits of sleep for EM consolidation were reduced in middle-aged adults (Backhaus et al., 2007) and older adults (Scullin, 2013; Cherdieu et al., 2014; Mander et al., 2014; Baran et al., 2016), whereas other studies have shown that sleep benefits are preserved in the elderly; for instance, one study revealed an attenuation of forgetting after sleep during a cued recall of word-pairs (Wilson et al., 2012) while another found that sleep protected the performance of a visuospatial memory task from interference (Sonni and Spencer, 2015). A possible explanation for this discrepancy in the effect of sleep in aging could be the use of different memory tasks to investigate EM consolidation in younger and older adults. Most of these tasks typically require the participant to learn a list of word-pairs or carry out an object-location task and then perform cued recall/recognition after a time interval (Gui et al., 2017). Hence, the different components of EM (factual, spatial, temporal, and details) and the ability to associate them *(binding performance)* are poorly investigated. One exception is the study by Rauchs et al. (2004) which investigated the relationship between EM consolidation and sleep deprivation in young adults through a What-Where-When task. In their learning task, 14 words were associated with two locations, the top or bottom of a sheet (spatial information), and assigned to two different lists (temporal information). Participants were asked to memorize each word and its spatio-temporal context, then consolidation was tested after 4-h retention intervals which followed sleep and occurred in either the first or the second half of the night. The data mainly demonstrated the benefit of different stages of sleep for consolidation of the spatio-temporal contextual information and the associated sense of autonoetic consciousness (Remember/Know paradigm, Tulving, 1985). In the same vein, van der Helm et al. (2011) employed a learning task which required associating items and their context and showed that, in younger adults, sleep preferentially benefited contextual aspects of episodic events. Unfortunately, no such study using this type of task has yet been undertaken in the elderly. Moreover, Aly and Moscovitch (2010) suggested that to assess sleep-dependent EM consolidation properly in the elderly, a more ecological approach could be a crucial factor. They evaluated the effect of sleep on EM in younger and older adults using naturalistic material such as stories or autobiographical episodes. The personal events to be remembered were assessed by means of 12 questions evaluating memory of the previous morning or evening in comparison with the recall of two stories learned from the Logical Memory section of the Wechsler Memory Scale III (WMS-III; Wechsler, 1997). The results indicated that while older adults performed less well than younger adults overall, interestingly memory consolidation of recent personal events as well as new stories benefited from a night of sleep compared to active wakefulness in both age groups. Accordingly, the authors suggested that sleep continues to enhance EM in aging especially when the to-be-remembered material engages interests close to the daily life of older adults.

Thus, the main aim of the present study was to evaluate the effect of daily life conditions on EM consolidation, through an ecological approach focused on the incidental encoding of everyday life-like events and the retrieval of associative aspects of EM performances in younger and older adults after a period favoring the consolidation process (sleep) vs. a period that does not favor it (active wake). We investigated whether sleep benefits memory in a What-Where-When task in older adults compared to younger adults.

To this end, we used Virtual Reality (VR) technology, which creates immersion in situations close to daily life with experimental control of What-Where-When information and perceptual details (Riva et al., 2007; Parsons and Rizzo, 2008; Abichou et al., 2017; Plancher and Piolino, 2017; La Corte et al., 2019). We assessed the effect of sleep vs. active wake on the binding of four aspects of naturalistic events, namely what, where, when and details. We analyzed the effect of sleep vs. active wake separately on each aspect (what, what-when, what-where, and what-details) to assess whether sleep specifically favors one aspect over another. We also evaluated the effect of sleep vs. active wake on recognition performances and the associated autonoetic consciousness (Remember/Know paradigm; Tulving, 1985).

First, whatever the interval type (Sleep or Active Wake), we predicted that younger adults would outperform older adults in binding performance (Aly and Moscovitch, 2010; Plancher et al., 2010). Second, we expected that the effect of sleep on the memory of naturalistic experience would be superior (i.e., reduce forgetting and enhancement) to the effect of active wakefulness (i.e., forgetting, or at best maintenance) (Aly and Moscovitch, 2010; Wilson et al., 2012). Lastly, we expected both age groups to benefit from sleep, but since sleep is reported to undergo deleterious age-related changes (Ohayon et al., 2004; Scullin, 2013), we expected that sleep would benefit younger adults more than older adults, especially concerning binding performance. In contrast, we expected that active wake would have a detrimental effect on binding performance, especially in older adults.

## Materials and Methods

### Participants

A total of 40 younger adults (22 ± 3 years) and 40 older adults (69 ± 5 years) took part in this study. Four younger adults were excluded because they were naturally short sleepers (<6 h), leaving a final group of 36 younger participants. Younger adults were recruited from Paris Descartes University and through flyers placed around the university. Older adults were recruited from the University of the Third Age at Paris Descartes University. They were paid at a rate of 10€/h or with course credit. This study was carried out in accordance with the ethics recommendations of Paris Descartes University and was approved by the local ethics committee of the Institute of Psychology at Paris Descartes University. All participants were informed of the academic nature of the study and gave their written informed consent for their participation in the study in accordance with the Helsinki Declaration.

We ensured that all participants had unimpaired or corrected-to-normal vision. None of them had any prior history of drug or alcohol abuse or neurologic, psychiatric, or sleep disorders. Participants were instructed to be drug, alcohol, and caffeine free for 24 h prior to and during the experiment. Participants were fluent in French, and were matched on their verbal IQ as assessed by the Mill Hill test (≥percentile 50, French translation; Deltour, 1993). None of them presented signs of depression based on the Beck Depression Inventory (BDI < 15; Hautzinger et al., 1995). To test for basic cognitive dysfunction in older adults, they were administered the Mini-Mental State Examination (MMSE > 26/30; Folstein et al., 1975) (Table 1).

**Table 1:** Participant characteristics: shown here are the means of demographic, inclusion, and neuropsychological measures across experimental groups.

	Active wake group		Sleep group		Age effects	Interval type effects	Interaction
					
	Younger adults	Older adults	Younger adults	Older adults	*F*(1,71)	*F*(1,71)	*F*(1,71)
**Demographic measures**							
Participants (M/F)	18 (5/13)	20 (10/10)	18 (10/8)	20 (7/13)			
Age *(yrs)*	23 (4.85)	70.30 (7.89)	22.05 (3.03)	69.20 (5.45)	1319.02	0.62	0.004
					***p* = 0.00**	*p* = 0.43	*p* = 0.95
					η^2^ = 0.95	η^2^ = 0.00	η^2^ = 0.00
Education^∗^	6.78 (0.55)	6.20 (1.10)	6.78 (0.73)	5.30 (1.66)	16.08	3.08	3.08
					***p* = 0.00**	*p* = 0.08	*p* = 0.08
					η^2^ = 0.18	η^2^ = 0.04	η^2^ = 0.04
**Inclusion test**							
Mill Hill	31.94 (4.77)	38.05 (4.20)	32.11 (5.93)	35.85 (6.15)	16.62	0.7	0.937
					***p* = 0.00**	*p* = 0.41	*p* = 0.34
					η^2^ = 0.18	η^2^ = 0.00	η^2^ = 0.02
BDI	3.33 (3.5)	2.10 (2.45)	4.2 (2.07)	3.70 (2.68)	1.9	3.75	0.25
					*p* = 0.17	*p* = 0.06	*p* = 0.62
					η^2^ = 0.02	η^2^ = 0.05	η^2^ = 0.00
MMSE	–	29.10 (1.02)	–	29.85 (0.98)	–	0.65	–
						*p* = 0.43	
**Neuropsychological measures**							
*Session 1*							
TMT *(s)*	33.44 (18.08)	62.15 (28.68)	31.17 (16.78)	68.05 (56.64)	16.88	0.05	0.26
					***p* = 0.00**	*p* = 0.82	*p* = 0.61
					η^2^ = 0.19	η^2^ = 0.00	η^2^ = 0.00
Digit span	16.61 (4.03)	13.90 (3.16)	15.83 (2.57)	13.30 (3.48)	11.6	0.80	0.01
					***p* = 0.001**	*p* = 0.38	*p* = 0.9
					η^2^ = 0.14	η^2^ = 0.01	η^2^ = 0.00
*Session 2*							
Family picture test (standard EM test)	–	33.25 (7.93)	–	32.25 (7.00)	–	0.17	–
						*p* = 0.68	
						η^2^ = 0.00	
What-Where-When span	9.33 (2.20)	5.75 (1.55)	9.50 (2.43)	5.95 (2.78)	46.25	0.12	0.00
					***p* = 0.00**	*p* = 0.73	*p* = 0.97
					η^2^ = 0.39	η_2_ = 0.00	η^2^ = 0.00
FAB	–	17.20 (0.70)	–	16.70 (1.26)	–	2.41	–
						*p* = 0.13	


*Values in parentheses represent standard deviations; *p*-values in bold represent significance.*

*^∗^Scale from 1 to 7 *(no schooling to graduate studies completed*).*

**FIGURE 1:**
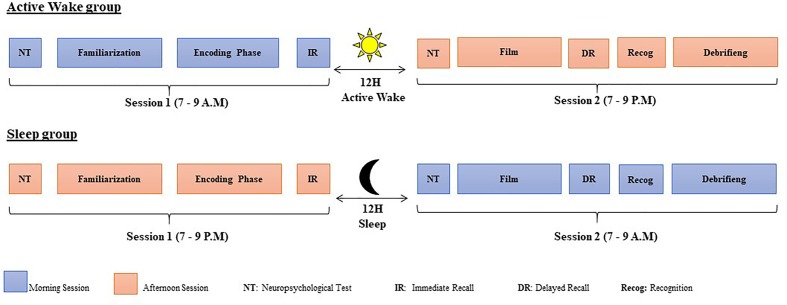
Experimental design.

Within each age group (young and older adults), participants were randomly assigned to either an Active Wake or a Sleep interval group and were individually tested (Figure 1). Participants in the Active Wake interval groups (18 younger adults and 20 older adults) performed the first session at 7–9 a.m., and then went about their normal daily activities outside the laboratory. Twelve hours later at 7–9 p.m., they were tested during the second session. Participants were instructed not to nap or consume alcohol during this time. In the Sleep interval groups, participants (18 younger adults and 20 older adults) performed the first session at 7–9 p.m. and were tested during the second session, 12 h later the following morning, after a full night’s sleep at 7–9 a.m. (see Debarnot et al., 2009, 2013, 2015 for a similar design). The first and second sessions did not exceed 1 h 30. None of the participants complained about the time or the duration of the experiment and they gave their informed consent to come back for the second session.

The sex ratio was equivalent across the four groups (χ^2^ = 3.7772, *p* = 0.28). To assess cognitive abilities across participants assigned to the four experimental conditions, they were screened using a brief battery evaluating executive functions (switching) by the Trail Making Test (Lezak et al., 2004), working memory by Digit forward/backward span (Wechsler, 1997) and basic binding performance using the multimodal what-where-when span (The House Test; Picard et al., 2012). For the older adults, additional standard tests of executive function, the Frontal Assessment Battery (FAB; Dubois et al., 2000), and EM, the Family Pictures test (FP, Wechsler, 2001) were administered (Table 1). In order to be relatively comparable to our virtual reality EM assessment (VREM test), we used the Family Picture test in which participants must learn a series of pictures and then recall the characters present in the scene, what each character did and where each of them was.

### Sleep Assessment

Sleep characteristics were assessed in the first session during recruitment via the Pittsburgh Sleep Quality Index (PSQI; Buysse et al., 1989) to assess sleep quality and quantity over the previous 30 days. This test was administered to exclude participants who were experiencing obvious disturbances during their sleep-wake cycles and to ascertain the participants’ predisposition to benefit from sleep for memory consolidation. None of the participants reported sleep disorders and none were taking medication that affected sleep architecture or the central nervous system. No extreme evening and morning type individuals or regular nappers were reported. Participants also completed the Stanford Sleepiness Scale (SSS) (Hoddes et al., 1973), a seven-point scale, with 1 being the most alert state. Lastly, the evaluation of sleep duration and waking behavior in the previous 24 h was evaluated by the St. Mary’s Hospital Scale (Ellis et al., 1981) (Table 2).

**Table 2:** Results of sleep measures: shown here are the mean questionnaire scores across experimental groups.

	Active wake group		Sleep group		Age effects	Interval type effects	Interaction
					
Sleep measures	Younger adults	Older adults	Younger adults	Older adults	*F*(1,71)	*F*(1,71)	*F*(1,71)
PSQI^∗^	5.39 (3.55)	5.75 (3.93)	6.67 (3.53)	6.80 (4.65)	0.08	1.75	0.02
					*p* = 0.78	*p* = 0.19	*p* = 0.9
					η^2^ = 0.00	η^2^ = 0.02	η^2^ = 0.00
St. Mary’s Hospital (sleep duration hrs) *^∗∗^*	7 (1)	7 (1.6)	6.3 (1.2)	7.15 (1)	2	0.82	2.37
					*p* = 0.16	*p* = 0.37	*p* = 0.13
					η^2^ = 0.02	η^2^ = 0.01	η^2^ = 0.03
SSS1^∗∗∗^	2.41 (0.9)	1.85 (1.04)	2.67 (0.84)	1.74 (0.87)	11.9	0.11	0.72
					***p* = 0.00**	*p* = 0.74	*p* = 0.39
					η^2^ = 0.15	η^2^ = 0.00	η^2^ = 0.01
SSS2	2.25 (0.77)	1.85 (0.93)	2.66 (.78)	1.74 (0.80)	10.25	0.4	1.42
					***p* = 0.00**	*p* = 0.53	*p* = 0.24
					η^2^ = 0.13	η^2^ = 0.00	η^2^ = 0.02


*Values in parentheses represent standard deviations; *p*-values in bold represent significance ^∗^**PSQI =** Pittsburgh Sleep Quality Index **^∗∗^St. Mary’s Hospital:** the night prior to encoding for the *Active Wake group* and between encoding and recall session for the *Sleep group*^∗∗∗^**SSS1** (session 1)/ **SSS2** (session 2) = Stanford Sleepiness Scale, 1 being the most alert.*

### Virtual Reality Episodic Memory (VREM) Assessment

#### The Virtual Apparatus

The virtual environment was created with Virtools Dev 3.0 software^1^ and in-house software (EditoMem, SimulaMem) to create a virtual town and associated events. It is a 3D computer model of an artificial environment presented on a PC laptop (15.6 inches; 34.5 cm × 19.5 cm) and projected 30 cm in front of the participant who is seated in a comfortable chair and navigates as a pedestrian in the virtual town using a joystick.

#### The Virtual Environment

**FIGURE 2:**
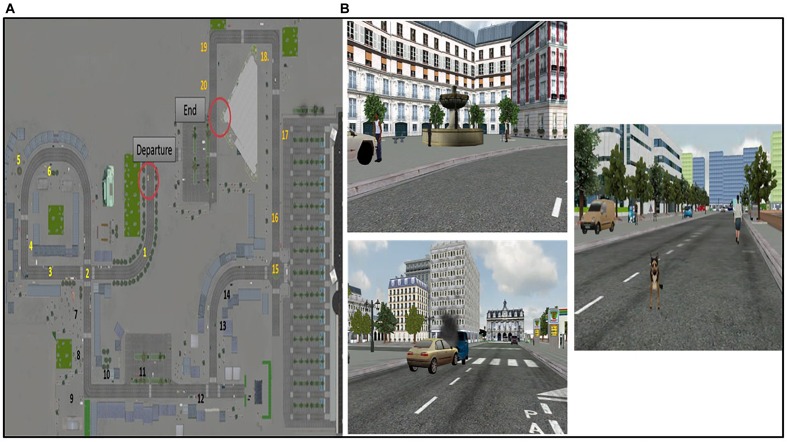
The virtual urban environment. **(A)** Topography of the virtual city on which the spatio-temporal location of events (items 1 to 20) is mentioned. **(B)** Example of events encountered during the navigation.

The virtual environment is a multimodal urban environment created from photos of Paris based on previously validated virtual reality cities used in aging studies (Plancher et al., 2010, 2012; Jebara et al., 2014; Plancher and Piolino, 2017). The participant is immersed in the virtual city, hearing sounds, seeing rich perceptual details and interacting thanks to his/her own displacement using a joystick. There is only one possible route through the virtual city, composed of 10 turns. The route is rich in objects and elements close to daily life to simulate a naturalistic urban environment (buildings, shops, people, trees, etc.), and contains 20 relevant unique events encountered during navigation (Figure 2A). Each event is related to a specific spatio-temporal context and specific perceptual details. For example, a white fountain with two levels and water flow is encountered at the beginning of the route on the left. There is an accident between a blue car and a gold car, which emit fumes, in the middle of the road straight ahead. A brown dog suddenly appears, barking, at the end of the pathway straight ahead (Figure 2B).

#### The What-Where-When VR Task

Using this virtual urban environment, we developed a What-Where-When VR task based on previously validated (VREM) tasks in normal aging (Plancher et al., 2010, 2012; Jebara et al., 2014; Plancher and Piolino, 2017; La Corte et al., 2019). This VR task used a series of naturalistic events embedded in the virtual environment to evaluate memory of the content of each scene (what), its perceptual details (details), the related temporal (when) and spatial (where) information as well as the binding of these features.

##### Familiarization phase

**FIGURE 3:**
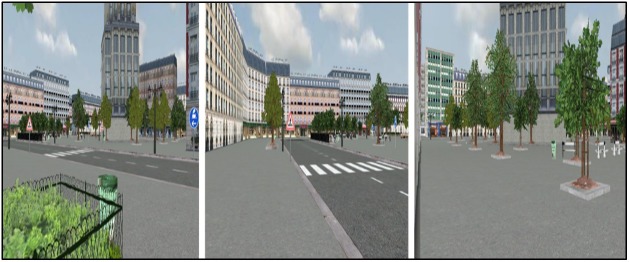
Training environment (familiarization phase).

Subjects underwent a training session in an environment devoid of relevant events and containing only general elements (e.g., building, trees, etc.) (Figure 3). They were free to navigate anywhere on the training track using a joystick. The training session lasted until they felt comfortable with the apparatus.

##### Encoding phase

Subjects were immersed in the VR environment, the light in the room was switched off in order to increase the immersion and sense of presence but also to ensure that all participants experienced the same room-condition. They were asked to visit the city and to pay attention to all the details in order to tell us afterward if they would recommend living in this city to a friend. They were also told that they would be asked to give an assessment of the virtual environment. The task involved incidental encoding as the participants were unaware that their memory would be tested afterward. The navigation lasted on average 10 min.

##### Retrieval phase

###### Immediate free recall

Five minutes after the encoding phase, subjects were asked to verbally report the maximum of events encountered during their navigation and the associated elements: *“Now, try to recall the maximum of unique elements and events that you remember having seen during your exploration of the virtual city from the beginning to the end. For each event, specify the maximum of* ***perceptive details*** *(for example colors, sounds), the* ***spatial position*** *(if the elements were on your right, left, or in front of you)*, ***the temporal position*** *(at the beginning, in the middle, or at the end of the exploration). As far as possible*, ***try to recall the items in chronological order***. *There are about 20 remarkable elements to remember in this city.”* No feedback was provided to participants, but at the end the experimenter asked them if they would like to add any spatio-temporal information or details about the remembered scenes. The experimenter noted all recalls on a structured response grid which had been validated in several previous VREM studies in our laboratory (Plancher et al., 2010, 2012, 2018; Jebara et al., 2014; Picard et al., 2017).

The accuracy of the recall of the what, where, when and perceptual details assigned to each of the 20 scenes was computed. We calculated a **binding score** (What-Where-When) to assess associative memory performance. We also computed a **high binding** **score** which took the association between perceptual details into account in addition (What-Where-When and Details). EM subscores (What, What-Where, What-When, and What-Details) were also computed. This evaluation enables the effect of consolidation among age groups and between the Active Wake and Sleep interval conditions on different types of binding to be assessed. In each case the maximum score was 20.

To take one of the above-mentioned examples, if the participant correctly reported having seen a car accident, one point was given for factual information (car accident, What score) and for each correct piece of associated information: spatial location (in front, What-Where score), temporal situation (halfway through the navigation, What-When score), perceptual details (blue and gold cars, fumes, etc., What-Details), for binding score (What-Where-When) and for high binding score (What-Where-When and Details). If the perceptual details were incorrectly recalled, but factual, spatial, and temporal contents were correct, each recall was scored 1, except for high binding which was scored 0 (for detailed scoring, see Supplementary Material).

###### Delayed free recall

During the second session (after 12 h), the participant began by watching a film “The mysteries of the cosmos: the sun king” during 9 min 50 s in order to ensure that delayed free recall was performed in the same context for each participant. The delayed free recall was carried out and scored in a similar manner to the first immediate free recall.

###### Recognition test

During the second session (after 12 h), and a few minutes after the delayed free recall test, each participant underwent a visual recognition test: a series of 35 stimuli with 20 old stimuli (snapshots from the virtual environment, stimuli that participants had already seen) and 15 new stimuli (snapshots from another virtual environment, 8 of which were semantically related to the environment already seen and 7 not related) was presented to the participants on the laptop and they had to decide which items they had seen during immersion in the virtual environment. Then, for each item recognized, they were requested to say whether they could mentally relive the spatio-temporal encoding context of the event or whether they just knew it (i.e., a remember versus a know judgment, Tulving, 1985).

We calculated the percentage of *correct recognitions* of factual information (What) (maximum 20), then, we computed the percentage of contextual information (What-Where, What-When) and the percentage of remembering judgments relative to correct factual recognition. We also computed the percentage of correct rejections of neutral (maximum = 7) and semantically related distractors (maximum = 8).

###### Debriefing

At the end of the experiment, subjects completed a self-administered questionnaire to evaluate their immersion, sense of presence in the virtual environment, navigation difficulties, and assessment of the environment (Table 3).

**Table 3:** Evaluation of the virtual environment: shown here are the means of Virtual Reality navigation duration and debriefing scores across experimental groups.

	Active wake group		Sleep group		Age effects	Interval type effects	Interaction
					
	Younger adults	Older adults	Younger adults	Older adults	*F*(1,71)	*F*(1,71)	*F*(1,71)
Navigation duration (s)	596.05 (110.17)	728.3 (187.76)	609.56 (132.56)	595.50 (107.10)	3.42	3.48	5.24
					*p* = 0.07	*p* = 0.07	***p* = 0.02**
					η^2^ = 0.05	η^2^ = 0.05	η^2^ = 0.07
Use of laptop^∗^	6.89 (0.32)	6.22 (1.93)	6.67 (0.84)	5.50 (2.42)	5.78	1.53	0.43
					***p* = 0.02**	*p* = 0.22	*p* = 0.51
					η^2^ = 0.08	η^2^ = 1.53	η^2^ = 0.00
**Debriefing^∗∗^**							
Navigation appreciation *(Q1)*****	7.67 (1.78)	7.20 (2.35)	7.05 (2.01)	7.80 (2.04)	0.04	0.00	1.83
					*p* = 0.84	*p* = 0.94	*p* = 0.18
					η^2^ = 0.00	η^2^ = 0.00	η^2^ = 0.03
Presence *(Q2, Q3, Q4)*	18.28 (6.6)	17.44 (8)	13.28 (6.26)	18.77 (8.29)	1.86	1.15	3.43
					*p* = 0.17	*p* = 0.29	*p* = 0.07
					η^2^ = 0.03	η^2^ = 0.02	η^2^ = 0.05
Task difficulty *(Q5, Q6)*	2.33 (1.19)	4.33 (3)	3.28 (2.35)	4.30 (2.8)	7	0.34	0.73
					***p* = 0.01**	*p* = 0.43	*p* = 0.4
					η^2^ = 0.09	η^2^ = 0.00	η^2^ = 0.01


*Values in parentheses represent standard deviations; *p*-values in bold represent significance.*

*^∗^Laptop frequency of use: scale from 1 (never used) to 7 (daily use).*

*^∗∗^Ratings were made on a 1 *(Strongly Disagree)* to 10 *(Strongly Agree)* point Likert scale.*

*Q1: Overall, did you enjoy your exploration of the proposed virtual environment? Q2: During the navigation in the virtual environment, did you have the feeling of walking in the city? Q3: Did you get the impression that the avatars were like real people? Q4: Did you feel as if you were really in the city? Q5: Did you find navigation in the virtual environment difficult? Q6: Did you find the recall task difficult?*

### Data Analysis

All the analyses were performed using *Statistica 13* software. A series of analyses of variance (ANOVAs) with **Age group** (Older adults vs. Younger adults) and **Interval type** (Active Wake vs. Sleep) were performed for neuropsychological evaluations, variables assessing sleep and debriefing scales. Concerning VREM assessment, we analyzed navigation duration at encoding and free recall performances for binding score (What-Where-When) and high binding score (What-Where-When-Details), then for each component (What, What-Where, What-When and What-Details) and finally, for recognition and remember judgment performances.

We first assessed the baseline difference between **Age group** (Young vs. Older) through **Interval type** (Active Wake vs. Sleep) via a series of ANCOVAs (controlling navigation duration) on performances at session 1. Then, to assess EM changes over active wake and sleep interval, a series of three-way **Session** (S1 vs. S2) × **Age group** (Young vs. Older) × **Interval type** (Active Wake vs. Sleep) ANCOVAs controlling for navigation duration was also performed. Finally, we analyzed the different recognition performances and debriefing scales in session 2 via a series of ANCOVAs controlling navigation duration with **Age group** (Older adults vs. Younger adults) and **Interval type** (Active Wake vs. Sleep). Each size effect (η^2^) is reported and when interaction was significant each pairwise comparison using PLSD Fisher *post hoc* tests was calculated. Results were considered significant at *p* < 0.05. Datasets and analyses are available on request from the authors.

## Results

### Neuropsychological Evaluations

Only a predictable age difference was revealed on each test, but no effect of Interval type (Wake vs. Sleep) or Age group × Interval type interaction was found. For each age group, the cognitive performances on tests assessing executive function, flexibility and working memory performances did not differ according to the Interval type. Older adults did not differ on standard tests assessing EM and frontal functions according to the Interval type (see Table 1).

### Sleep Assessment

The main effect of Age on the amount of sleep overnight using the St. Mary’s Hospital scale was not significant. Similarly the effect of interval type and the Age group × Interval type interaction were not significant. Subjective sleep quality assessed by the PSQI did not differ across Age groups and interval type and no Age group × Interval type interaction was found. Subjective measures of alertness and sleepiness assessed during the two sessions via SSS1 and SSS2 scales revealed a main effect of Age. Younger participants reported being less alert than older adults for both Interval types (Active Wake vs. Sleep). However, no effect of Interval type or Age group × Interval type interaction was significant for either session. Thus the two age groups were well-matched for sleep measures across interval type (Active Wake vs. Sleep) (see Table 2).

### Navigation Duration and Debriefing Scales

Concerning navigation duration, no effect of Age and Interval type was found. However, we observed an Age group × Interval type interaction. As the *post hoc* test indicated that older adults in the Active wake group spent more time navigating than older adults in the sleep group, navigation duration was controlled for in the following analyses. As regards the use of the laptop and task difficulty, we found an Age effect (*p* < 0.05). However, no effect of Interval type or Age group × Interval type interaction was found. Younger adults reported using laptops more frequently and task was reported to be easier than for older adults. No effect of Age and Interval type or Age group × Interval type interaction was found for navigation appreciation and sense of presence (see Table 3).

### Performances on Virtual Reality Episodic Memory (VREM) Assessment

#### Baseline Difference in Episodic Memory Performance

A preliminary check of initial performance at encoding (session 1) across interval type (Active Wake vs. Sleep) and Age group (Older adults vs. Younger adults) indicated an expected Age effect on **binding score** (What-Where-When) [*F*(1,71) = 12, *p* < 0.0001, η^2^ = 0.14] and **high binding score** (What-Where-When-Details) [*F*(1,71) = 26.56, *p* < 0.0001, η^2^ = 0.27]. However, there was no main effect of Interval type on binding [*F*(1,71) = 2, *p* = 0.16, η^2^ = 0.03] and high binding scores [*F*(1,71) = 1.52, *p* = 0.22, η^2^ = 0.02], and no effect of Age group × Interval type interaction on binding score [*F*(1,71) = 0.04, *p* = 0.83, η^2^ = 0.00] and high binding score [*F*(1,71) = 0.60, *p* = 0.44, η2 = 0.00].

An expected Age effect was revealed for all the **EM subscores**: What [*F*(1,71) = 22, *p* < 0.0001, η^2^ = 0.23], What-Where [*F*(1,71) = 23.46, *p* < 0.0001, η^2^ = 0.25], What-When [*F*(1,71) = 10.90, *p* < 0.01, η^2^ = 0.13] and What-Details [*F*(1,71) = 48.22, *p* < 0.0001, η^2^ = 0.40]. Importantly, there was no main effect of Interval type: What [*F*(1,71) = 0.01, *p* = 0.92, η^2^ = 0.00], What-Where [*F*(1,71) = 0.54, *p* = 0.50, η^2^ = 0.00], What-When [*F*(1,71) = 1.37, *p* = 0.24, η^2^ = 0.02 ] and What-Details [*F*(1,71) = 0.03, *p* = 0.86, η^2^ = 0.00]. Finally, there was no Age group × Interval type interaction: What [*F*(1,71) = 1.66, *p* = 0.20, η^2^ = 0.02], What-Where [*F*(1,71) = 1.51, *p* = 0.22, η^2^ = 0.02], What-When [*F*(1,71) = 0.09, *p* = 0.77, η^2^ = 0.00 ] and What-Details [*F*(1,71) = 0.4, *p* = 0.53, η^2^ = 0.00].

In sum, younger adults performed better than older adults at the encoding session whatever the interval type, the participants for each age group were well-matched across interval type and no difference in performances occurred depending on when the encoding was performed (in the morning or in the evening).

#### Episodic Memory Performance Over Active Wake and Sleep Intervals

For both age groups, EM recalls were diminished in the delayed recall relative to the immediate recall in the Active Wake interval and interestingly, they were enhanced following a sleep interval.

**FIGURE 4:**
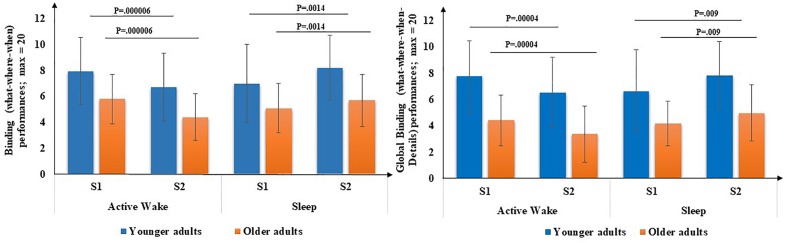
Binding performance (number of What-Where-When and What-Where-When-Details associations) through Interval type (Active Wake vs. Sleep) across Age group (Younger vs. Older). Error bars represent standard errors of the mean. NB: for reasons of readability, the effect of age is not reported here.

First, for both **binding scores** (Figure 4), there was a main effect of age [What-Where-When [*F*(1,71) = 16.71, *p* < 0.001, η^2^ = 0.19] and What-Where-When-Details [*F*(1,71) = 32.2, *p* < 0.001, η^2^ = 0.30], but no effect of Interval type [*F*(1,71) = 0.20, *p* = 0.65, η^2^ = 0.003] and [*F*(1,71) = 0.53, *p* = 0.47, η^2^ = 0.00], respectively, and no effect of Session [*F*(1,71) = 1.3, *p* = 0.26, η^2^ = 0.02] and [*F*(1,71) = 0.20, *p* = 0.68, η^2^ = 0.00], respectively. An interval type × Session interaction was revealed [*F*(1,71) = 34.8, *p* < 0.001, η^2^ = 0.33] and [*F*(1,71) = 33.72, *p* < 0.001, η^2^ = 0.32], respectively but no Age × Session interaction was found [*F*(1,71) = 1.31, *p* = 0.25, η^2^ = 0.02] and [*F*(1,71) = 0.07, *p* = 0.8, η^2^ = 0.00], respectively, and no three-way Age group × Interval type × Session interaction was found [*F*(1,71) = 0.09, *p* = 0.76, η^2^ = 0.00] and [*F*(1,71) = 0.54, *p* = 0.46, η^2^ = 0.00], respectively. *Post hoc* PLSD tests performed on Interval type × Session interaction indicated that, for both age groups, delayed recall performances of binding (What-Where-When) and high binding (What-Where-When and details) were significantly lower following an Active wake interval (*p* < 0.001) and significantly enhanced following sleep (*p* < 0.01).

**FIGURE 5:**
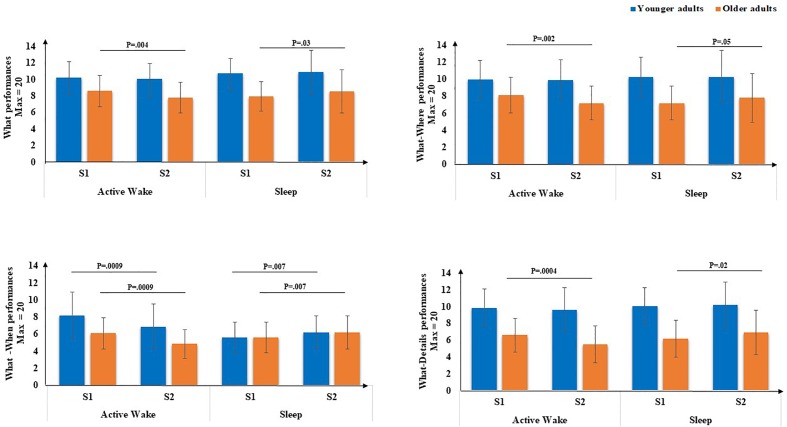
EM subscores (What, What-Where, What-When, and What-Details) through Interval type (Active Wake vs. Sleep) across Age group (Younger vs. Older). Error bars represent standard errors of the mean. NB: for reasons of readability, the effect of age is not reported here.

Second, concerning the **subscores** (Figure 5), there was a main effect of age for the **What score** [*F*(1,71) = 20.66, *p* < 0.001, η^2^ = 0.22], but no main effect of Interval type was found [*F*(1,71) = 0.45, *p* = 0.5, η^2^ = 0.00]. There was no Age × Session interaction [*F*(1,71) = 0.18, *p* = 0.67, η^2^ = 0.00], but a type × Session interaction was revealed [*F*(1,71) = 8.76, *p* < 0.01, η^2^ = 0.11], as well as an Age group × Interval type × Session [*F*(1,71) = 4.87, *p* < 0.5, η^2^ = 0.06]. *Post hoc* PLSD tests performed on Age group × Interval type × Session indicated that, for older adults, delayed free recall performances for the What subscore were significantly lower following Active Wake (*p* < 0.01) and significantly enhanced following sleep (*p* < 0.05). For younger adults, the decrease in delayed free recall performances during active wake interval and their enhancement following the sleep interval did not reach significance; there was no difference between sleep and active wake (*p* = 1).

Concerning **What-Details and What-Where EM subscores,** there was a main effect of age [*F*(1,71) = 46.18, *p* < 0.001, η^2^ = 0.39] and [*F*(1,71) = 21.35, *p* < 0.001, η^2^ = 0.23], respectively, but no effect of Interval type [*F*(1,71) = 0.70, *p* = 0.40, η^2^ = 0.00] and [*F*(1,71) = 0.01, *p* = 0.9, η^2^ = 0.00], respectively, and no effect of Session [*F*(1,71) = 0.31, *p* = 0.58, η^2^ = 0.00] and [*F*(1,71) = 0.23, *p* = 0.63, η^2^ = 0.00], respectively. Moreover, no Age × Session interaction was found for the two subscores [*F*(1,71) = 0.27, *p* = 0.60, η^2^ = 0.00] and [*F*(1,71) = 0.23, *p* = 0.62, η^2^ = 0.63], respectively, but an Interval type × Session interaction was found [*F*(1,71) = 13.46, *p* < 0.001, η^2^ = 0.16 ] and [*F*(1,71) = 6.7, *p* < 0.05, η^2^ = 0.08], respectively. Also, a three-way Age group × Interval type × Session interaction was found [*F*(1,71) = 5.75, *p* < 0.05, η^2^ = 0.07] and [*F*(1,71) = 4.2, *p* < 0.05, η^2^ = 0.05], respectively. *Post hoc* PLSD tests performed on Age group × Interval type × Session indicated that, for older adults, delayed free recall performances for these two sub-scores were significantly poorer following Active Wake (*p* < 0.001 and *p* < 0.01, respectively) and significantly enhanced following sleep (*p* < 0.05 and *p* = 0.050, respectively). For younger adults, the decrease in delayed free recall performances during the active wake interval and their enhancement following the sleep interval did not reach significance; there was no difference between sleep and wakefulness regarding What-Details and What-Where (*p* = 1).

Concerning the **What-When** **subscore**, there was a main effect of age [*F*(1,71) = 14.82, *p* < 0.001, η^2^ = 0.17], but no effect of Interval type [*F*(1,71) = 0.42, *p* = 0.52, η^2^ = 0.00], no effect of Session [*F*(1,71) = 0.96, *p* = 0.33, η^2^ = 0.01] and no Age × Session interaction [*F*(1,71) = 0.42, *p* = 0.52, η^2^ = 0.00] or three-way Age group × Interval type × Session interaction [*F*(1,71) = 0.15, *p* = 0.70, η^2^ = 0.00]. However, an Interval type × Session interaction was revealed [*F*(1,71) = 24.94, *p* < 0.001, η^2^ = 0.26]. *Post hoc* PLSD tests indicated that for both age groups, performances on delayed What-When free recall were significantly poorer following active wake (*p* < 0.001) and significantly enhanced following sleep (*p* < 0.01).

In sum, on the one hand, for older adults, all types of EM aspects as well as both binding performances were significantly poorer after the active wake interval and significantly enhanced after a sleep interval. For younger adults, binding performances and more especially factual-temporal associations were significantly poorer after an active wake interval and significantly enhanced after sleep.

#### Recognition Performance

Results and analyses are presented on Table 4. Results of percentage of **correct recognition** of factual (what) and correct contextual associated information (What-Where, What-When) indicated a main effect of Age, with younger adults showing better recognition performances than older adults, while there was no effect of Interval type nor Age group × Interval type interaction.

**Table 4:** ANCOVA results for recognition performances: shown here are the percentages of correct recognition of factual information (What), contextual information (What-Where, What-When) and Remember judgments (R) correctly associated to factual recognition and the percentage of correct rejections of neutral and semantically related distractors.

	Active wake group		Sleep group		Age effects	Interval type effects	Interaction
					
	Younger adults	Older adults	Younger adults	Older adults	*F*(1,71)	*F*(1,71)	*F*(1,71)
**Hits correct responses %**							
What recognition %	73 (11.78)	51 (15)	68.6 (18.7)	48.25 (21)	**33.75**	0.43	0.78
					***p* = 0.00**	*p* = 0.51	*p* = 0.38
					η^2^ = 0.32	η^2^ = 0.00	η^2^ = 0.01
What-Where recognition %	63 (12)	43.75 (14.5)	60.9 (19)	41.25 (21)	28	0.11	0.39
					***p* = 0.00**	*p* = 0.74	*p* = 0.53
					η^2^ = 0.28	η^2^ = 0.00	η^2^ = 0.00
What-When recognition %	45 (12.7)	26.5 (12)	42.5 (16)	29 (15)	24.6	0.00	0.73
					***p* = 0.00**	*p* = 0.99	*p* = 0.4
					η^2^ = 0.25	η^2^ = 0.00	η^2^ = 0.01
**R judgment correctly associated to what %**	53 (15)	45 (15)	54 (20)	52 (17)	1.05	0.42	0.0
					*p* = 0.31	*p* = 0.52	*p* = 1
					η^2^ = 0.01	η^2^ = 0.00	η_2_ = 0.00
**Distractor correct rejection %**	
Neutral %	90 (11)	92 (17)	97.7 (7)	88.6 (16)	1.35	0.32	2.76
					*p* = 0.25	*p* = 0.6	*p* = 0.1
					η^2^ = 0.02	η^2^ = 0.00	η^2^ = 0.04
Semantically associated %	82.7 (17)	93.8 (8.6)	95 (7.5)	89.4 (14)	0.94	1.8	8.6
					*p* = 0.33	*p* = 0.18	***p* = 0.00**
					η^2^ = 0.01	η^2^ = 0.02	η^2^ = 0.11


*Values in parentheses represent standard deviations; *p*-values in bold represent significance.*

When computing the **percentage of Remember** judgments relative to correct factual recognition for each participant, we no longer found any effect of age group, interval type or Age group × Interval type interaction.

Concerning the **good rejection of neutral distractors**, the ANCOVA revealed no effect of Age, no effect of Interval type and no Age group × Interval type interaction. However, for **good rejection of semantically** **related distractors**, the ANCOVA revealed no effect of age or Interval type but an Age group × Interval type interaction. The *post hoc* PLSD tests indicated that younger adults had a better correct rejection of semantically related distractors after a sleep interval than older adults (*p* < 0.05).

## Discussion

In the present study, a naturalistic (What-Where-When) EM task, rich in details and spatio-temporal context, was implemented in a virtual environment and was used to evaluate the effect of extended overnight sleep vs. extended active wakefulness on the consolidation of personally experienced events close to daily situations, in younger and older adults. As expected, the findings showed an age-related decline in EM performance for older adults compared to their younger counterparts, but most importantly, they revealed for both age groups a decline in memory performances following a period of active wakefulness and enhancement following sleep. We will briefly discuss the age-related effects on EM functioning and consider forgetting performances following the wakefulness condition, then we will focus on the effect of sleep on EM consolidation.

### Age-Related Decline of Naturalistic What-Where-When EM Task

Basically, it is assumed that age-related differences in contextual memory are greater than those in memory for content (Chalfonte and Johnson, 1996; Naveh-Benjamin, 2000; Kessels et al., 2007; Cheke, 2016). The retrieval performances from our incidental encoding session indicate that younger adults performed better than older ones at recalling contextual information (what-details, what-where, and what-when associations) and especially feature binding (i.e., what-where-when and what-where-when and details associations), but also better at recalling factual information. This pattern of performance was corroborated during the second session (12 h later) regardless of the Interval type (Active Wake or Sleep). Since recall of factual information is sensitive to the attention allocated during encoding and the amount of effort required during retrieval (Spencer and Raz, 1995), the use of incidental encoding and free recall may account for age-related deficits of factual information found in our study. Nevertheless, this age-related decline for factual information was also observed via recognition, which may indicate genuine incidental encoding deficits in older adults. However, when computing the percentage of remembering judgments relative to factual information, the effect of age on recognition disappeared, indicating quantitative rather than qualitative differences between younger and older adults for correct memory.

It should be mentioned that during the debriefing, older adults more frequently reported that the navigation was difficult compared to their younger counterparts; nevertheless, both age groups manifested an equivalent sense of presence in the virtual environment and their appreciation of the navigation was similar. The results of binding performance are in agreement with previous findings, suggesting an impaired binding in aging (Chalfonte and Johnson, 1996; Kessels et al., 2007), using a real-world What-Where-When memory task (Mazurek et al., 2015) or virtual What-Where-When EM tasks (Plancher et al., 2012; Jebara et al., 2014). This deficit may be possibly related to diminished activation of the hippocampus and changes in the activity of the prefrontal cortex (Mitchell et al., 2000; Hedden and Gabrieli, 2004). Interestingly, we previously showed that binding deficits in aging were independent of the incidental or intentional encoding of naturalistic scenes presented in virtual environments (Plancher et al., 2010), but that active encoding thanks to self-initiated activity in the virtual world (sensorimotor or decisional activity) enhanced long-term feature binding (Plancher et al., 2012; Jebara et al., 2014). In the same line, using a laboratory What-Where-When EM task (“treasure-hunt task”), Cheke (2016) showed that older adults can improve their binding by using self-initiated encoding strategies.

Thus, our results confirm a noticeable decline of EM in the elderly reported in several studies (Cabeza et al., 2000; Naveh-Benjamin, 2000; Glisky et al., 2001; Naveh-Benjamin et al., 2003; Kessels et al., 2007; Wang, 2016), but extend it to an ecological situation via incidental active encoding of naturalistic experience in virtual reality where different aspects relevant to EM and its associated phenomenology were assessed (for a review see, Abichou et al., 2017; Plancher and Piolino, 2017; La Corte et al., 2019). Most importantly, our study pointed out a general age-related impairment affecting different components of EM as well as their related binding.

### Dynamic of EM Free Recall Through Active Wake vs. Sleep Interval

For both age groups, EM free recall was diminished in the delayed recall relative to the immediate recall in the Active Wake interval while it was strengthened following a sleep interval. This pattern can not be attributed to some confounding effects, as participants were well-matched according to the type of interval on basic neuropsychological performance and sleep measure. In addition, we checked for the elderly that there was no difference concerning standard EM assessment, depending on when the test was done (in the morning or in the evening).

#### The Effect of Active Wake on EM Consolidation

When evaluating the effect of active wake on EM retention, our data from delayed free recall highlight a significant forgetting following the Active Wake interval for both age groups. All types of information (i.e., factual, contextual, and details associations) and their related binding were affected by forgetting in older adults, but it only concerned the association of factual and temporal information in younger adults. These findings support the idea of Active Wake as an unfavorable period for consolidation (Craig et al., 2016; Craig and Dewar, 2018). During Active Wake participants were engaged in their daily activities outside the laboratory (e.g., taking classes at the university), which may involve encoding new information or experiences and thus create retrospective interference (Dewar et al., 2007; Wamsley, 2019).

Retrospective interference is an explanation for forgetting in long term memory while memory consolidation is understood as a process increasing resistance to interference rather than permitting performance enhancement (Ellenbogen et al., 2006b; Mednick et al., 2009, 2011). In this line, according to the **Opportunistic hypothesis** (Mednick et al., 2011), neural representations are vulnerable to the interfering effects of new learning. At a cellular level, new hippocampal LTP induction can interfere with the maintenance of older LTP. Besides, subsequently encoded memories can compete for the same neural pathway that was used to consolidate previously encoded information.

However, this deleterious effect of Active Wake on free recall disappeared on recognition and remember judgments for both age groups. This finding may suggest that the memory trace was still present after a period of active wakefulness (about 12 h), but less spontaneously accessible in free recall, maybe because of a reduction in executive functions at the end of the day. Alternatively, it may indicate that active wake did not protect against forgetting, but rather that our recognition task was less sensitive to detect deficits than delayed free recall.

#### The Effect of Sleep on EM Consolidation

When investigating the effect of sleep on EM retention, our results from free recall showed that for both age groups, sleep compared to active wakefulness enhanced feature binding which is one of the main characteristics of EM.

*As regards young adults*, our findings indicated a significant enhancement in binding performance following sleep (both What-Where-When and What-Where-When and Details), and more specifically regarding temporal information (what-when association). One possible explanation might be that enhancement concerned the performance that was less effective in the first session (i.e., temporal information compared to factual, spatial and details information in younger adults, and each component in older ones). This is in line with studies that have shown for different domains of memory that sleep preferably consolidates weak rather than strong traces, and that it selectively provides maximum benefits for traces that proved to be most difficult prior to sleep (Kuriyama et al., 2004; Diekelmann et al., 2009). Otherwise, the specific benefit for temporal information in younger adults may indicate that remembering when events occur is a key feature for EM as this memory system relies on temporal projection into the past and the future (Wheeler et al., 1997; Tulving, 2002).

The consolidation of temporal information appears therefore crucial in long-lasting EM. This is in keeping with the role of sleep that has been found to favor the replay of new temporal information in a forward direction, which strengthens its integration to the EM trace in young adults (Drosopoulos et al., 2007; Griessenberger et al., 2012). This replay process is dependent on the hippocampus (CA3 network and dentate gyrus, Drosopoulos et al., 2007) and it has been suggested that it is related to a specific balance of REM and NREM sleep stages (Griessenberger et al., 2012).

In addition, the enhancement of binding in younger adults is in keeping with Rauchs’ study (Rauchs et al., 2004) which showed a benefit of sleep on the intentional recall of the spatial-temporal context associated with factual information using a What-Where-When task consisting of words associated with both spatial and temporal information. Moreover, our result is also in line with a more recent study (van der Helm et al., 2011), which revealed that for younger adults, sleep *(a nap period)* compared to daytime active wake selectively strengthens hippocampal-dependent aspects of declarative memory by promoting the retention of contextual EM characteristics relative to item memory. Those benefits were specifically correlated with stage 2-NREM. Here, we extended the finding to memory of naturalistic specific events and high binding performance which is in line with previous studies arguing that sleep reactivates item-context binding and strengthens the connection between item and context, supporting their redistribution into cortical regions where they are more stable (Drosopoulos et al., 2007; Inostroza and Born, 2013).

*About older adults*, our study is the first to investigate the effect of sleep on the elderly using a What-Where-When task and the first to evaluate the effect of sleep vs. active wakefulness on binding performance in naturalistic situations. We show, remarkably, a general enhancement following a sleep interval in all associative information (what-details, what-where, and what-when) as well as an enhancement in binding (what-where-when) and high binding performance (What-Where-When-Details). The present findings are in line with some previous studies that demonstrated the preservation of sleep benefits on declarative memory in aging (Aly and Moscovitch, 2010; Wilson et al., 2012; Sonni and Spencer, 2015). In contrast, they argue against other studies that failed to reveal any sleep benefit in aging (Scullin, 2013; Cherdieu et al., 2014; Mander et al., 2014; Baran et al., 2016). For instance, using a 3D spatial maze navigational task, one study showed that older adults did not exhibit any improvement in spatial memory after sleep, unlike younger ones. The decline was correlated to SWS activity changes related to aging (Varga et al., 2016). In our study, even if the spatial memory performances (i.e., the egocentric frame of reference) of older adults were lower than those of the younger adults, their spatial recall was enhanced after a night of sleep. Our result may be explained by the reliance of our participants on egocentric spatial information which is reported to be relatively preserved in aging compared to allocentric spatial information (Plancher et al., 2012; Colombo et al., 2017).

We may also suggest that studies using somewhat simple EM tasks (e.g., only factual information) or too remote from the interests of the elderly fail to demonstrate the effect of sleep while other studies using more complex EM tasks (e.g., factual and context) or a more naturalistic approach are able to reveal the benefit of sleep in aging (e.g., Aly and Moscovitch, 2010). Our results seem to confirm this assertion. In virtual environments, as in daily life, episodes are personally experienced (e.g., embodied and emotional experience) within a rich and specific spatio-temporal context (Plancher et al., 2012; Jebara et al., 2014; Colombo et al., 2017), which may make them more relevant for participants than more traditional laboratory material (e.g., association of word pairs). As suggested by Aly and Moscovitch (2010), this may increase the likelihood of their reactivation during sleep, engaging a deeper neural network.

Indeed, the hippocampus plays a crucial role in binding together different components of the new memory representation, giving rise to a multimodal representation which includes the conceptual, perceptual, and emotional components of the experience (Dudai, 2012; Josselyn et al., 2015). Theories of sleep-dependent consolidation (Giuditta et al., 1995; Gais, 2004; Rasch and Born, 2013) argue that sleep mediates the reactivation of those representations in the hippocampal networks, which favors the strengthening of their connection with cortical areas and mediates their gradual transfer to long-term store in the neocortex (Ts’o et al., 1986; Rasch and Born, 2013; Antony and Paller, 2017). This process is considered to result in an enhancement of memory recall performance of factual, contextual and details information, as well as their related binding following sleep. In fact, our findings suggest that the benefit of sleep on newly personally encoded events which is expected to rely on the role of the hippocampus is still efficient in normal aging, at least when memory traces are close to the daily life interests and personal relevance of the elderly.

### Recognition vs. Free Recall Performance Following Sleep Interval

Most noteworthy for our purpose, no effect of sleep was found in correct factual recognition and associated spatio-temporal context and remember judgments whatever the age group. However, we noted a beneficial effect of sleep in younger adults for the rejection of semantically related distractors, which is consistent with the fact that following sleep, enhancement of details and spatio-temporal recalls may lead to improved discrimination between the studied items and hence improved source memory (Johnson et al., 1993; Drosopoulos et al., 2007). A contrast between the positive effect of sleep on free recall and its absence of effect on recognition has already been reported in the literature. For instance, one study showed in younger adults a benefit of sleep with free recall but no effect with recognition (Rauchs et al., 2004). It was suggested that their recognition task (forced choice) was less sensitive to detect sleep benefits than the delayed free recall task. Additionally, in our case, recognition was performed a few minutes after the delayed free recall, so some ceiling effects can be assumed. This explanation is mentioned in other studies which consider that sleep benefits are more consistently revealed with recall than with recognition procedures (Koulack, 1997; Wagner et al., 2007; Diekelmann et al., 2009). These results suggest that sleep acts on memory consolidation by increasing the spontaneous accessibility to the memory trace which is highly involved during free recall. Thus, sleep may promote the integration of the newly acquired trace to pre-existing networks which reinforces it and facilitates its spontaneous access. Interestingly, studies reporting the benefits of sleep on EM consolidation in the elderly (our study and that by Aly and Moscovitch) used free recall and are the only ones that reported a benefit of sleep in the sense of enhancement, while the studies using cued free recall (word pair and a visuospatial learning task) reported rather an attenuation of forgetting or a stabilization (Wilson et al., 2012; Sonni and Spencer, 2015).

### Strengths, Limitations, and Future Avenues of Research

Overall, the present study has the merit of directly comparing (i) the effect of an extended period of overnight sleep and active wakefulness on memory for naturalistic experience, and (ii) the performance of young and older adults on a What-Where-When task. Moreover, the present findings are based on a virtual reality version of a What-Where-When VR task rich in perceptual details which allowed us to measure in a more ecologically valid way the hallmarks of EM (incidental encoding of events, the long-term associative memory of factual with perceptual details and spatiotemporal context, free recall, and sense of remembering). This advantageously gives a controlled measure of EM, and is enjoyable for young and older people. This is important since previous studies showed that performance in this type of virtual What-Where-When task correlates with the memory complaints in everyday life of older people, while a standard EM test does not (Plancher et al., 2010, 2012), and can better discriminate normal aging from mild cognitive impairment than standard EM tests (Plancher et al., 2012; Plancher and Piolino, 2017; La Corte et al., 2019).

Nevertheless, the study has some limitations. First, we did not use a fully immersive and interactive device, thus our results could be different with other material such as a VR head-mounted-display (HMD) or treadmill, especially for the active wakefulness condition (Tuena et al., 2017; La Corte et al., 2019). Second, to substantiate the present findings further studies should compare the results from our What-Where-When virtual reality task with other laboratory What-Where-When tasks (e.g., Cheke, 2016) to confirm the generality of the present effect of sleep on factual, location, and temporal information, as well as bound what–where–when information. Moreover, it would be important for future research to test whether What-Where-When VR EM measures are better than standard EM measures and other types of What-Where-When measures (Cheke and Clayton, 2013).

Regarding the specific role of sleep, the present study cannot determine whether the observed EM benefits using a naturalistic What-Where-When task are linked to a specific stage of sleep (**Unique-to-Sleep consolidation hypothesis**, Stickgold, 2005; Ellenbogen et al., 2006a; Diekelmann and Born, 2010) or rather to sleep as a period of low interference or reduced sensory input and cognitive load (**Opportunistic hypothesis**, Hasselmo and McClelland, 1999; Mednick et al., 2011). Our results suggest an enhancement of EM performance which is predicted by the **Unique-to-Sleep Consolidation Hypothesis** (Active Hypothesis). However, it would be interesting in the future to directly compare the effect of extended active vs. quiet wakefulness (i.e., high vs. low interference) and night sleep on EM consolidation to conclude on this issue. According to the **Opportunistic hypothesis**, it is expected that an extended post-learning period of quiet wakefulness has the same benefit as night sleep on protecting long-term feature binding of a naturalistic experience from interference, whereas according to the **Unique-to-Sleep consolidation hypothesis**, it is predicted that sleep is the only way to enhance EM performance.

## Conclusion

The present study suggested that, compared to a post-learning period of daytime active wakefulness, a post-learning period of overnight sleep not only protects against forgetting but can induce actual improvements in memory for a naturalistic experience. Most interestingly, the study showed that despite EM decline in elderly compared to younger adults, older adults still benefit from night sleep in supporting the consolidation of EM what-where-when and perceptual details components and their binding. Thus, the most important message is that sleep may still sustain efficient EM consolidation in healthy elderly people, at least concerning spontaneous accessibility to content memory and its context after a delay of 12 h and when the participant does not report sleep problems or disorders. Further studies should investigate the effect of sleep on long-lasting EM consolidation and the neural underpinning of this benefit of sleep in the elderly. This may enable future research to establish whether this profit on memory is only attributable to the special status of sleep or more generally to the presence of an extended post-learning period of reduced interference that may also concern quiet wakefulness.

## Author Contributions

KA and PP contributed to the conception and design of the study. EO and AG-B created the virtual environment. NH and KA contributed to the acquisition of the data. KA and PP organized the database and performed the statistical analysis. SN and VLC provided suggestions in the different drafts of the present manuscript. All the authors have read and approved the submitted version.

## Conflict of Interest Statement

The authors declare that the research was conducted in the absence of any commercial or financial relationships that could be construed as a potential conflict of interest.

## Footnotes

1 www.virtools.com
