# Supplementary material for: Young and Older Adults Benefit From Sleep, but Not From Active Wakefulness for Memory Consolidation of What-Where-When Naturalistic Events
Source: Front Aging Neurosci. 2019 Mar 20;11:58. doi: 10.3389/fnagi.2019.00058 (PMC6435496; doi:10.3389/fnagi.2019.00058)
Supplement: Supplementary file 1 [file Data_Sheet_1.docx]

**Young and older adults benefit from sleep, but not from active wakefulness for memory consolidation of What-Where-When naturalistic events**

**Supplementary material**

**Abichou et al., Frontiers in Aging Neuroscience, 2019**

1. **List of elements encountered in the virtual environment and their associated details and spatio-temporal context.**

Based on a pilot study (N= 70 younger adults) and previously published studies in aging (Jebara et al., 2014; Plancher et al., 2010, 2012)

| Elements (What) | Temporal information (When) | | Spatial information (Where) | Details  (some possible examples) |
| --- | --- | --- | --- | --- |
| 1. Barrier /downhill car | | Beginning | Ahead | A yellow car / black iron gates blocking the street |
| 1. Flower pot | | Beginning | Ahead | Green plant / fern / falling making noise |
| 1. A Car coming toward participant | | Beginning | Ahead | Blue car |
| 1. Removal van + cardboard boxes | | Beginning | On the right | White removal van /many cardboard boxes (N=4) with red tags /scattered on the street |
| 1. Fountain | | Beginning | On the left | Oval/ two level / three jets /sound of flowing water … |
| 1. Tricycle | | Beginning | On the right | Blue, Yellow and green / for children / in the road |
| 1. Play area | | Middle | On the right | Wooden multicolored play area/ Slide, swing/ no kids around… |
| 1. Boxers | | Middle | On the right | A black and a white man / each plays on his side / white top /red gloves/ fight… |
| 1. Train station | | Middle | On the right | Old station / with columns /heard a message announcing a departure … |
| 1. Dancers | | Middle | On the left | A white and a black man /music/ hip hop/break dance … |
| 1. A Man walking around | | Middle | On the left | Wearing a grey jacket /red shirt … |
| 1. Accident | | Middle | Ahead | Gold and blue cars /fumes … |
| 1. Post office /queue | | Middle | On the right | Large building /cash machine /men queuing |
| 1. A Baby in a car | | Middle | On the right | Black car / sound of baby crying / picture of a baby |
| 1. Roundabout | | End | Ahead/on the right | Flower, green area /wire fence… |
| 1. A dog | | End | Ahead | Brown and black / it is barking |
| 1. Newsstand | | End | On the right | A typical old Parisian kiosk/pointed roof/newspapers hanging on the side … |
| 1. Men on a ladder | | End | On the left | Black man /climbing … |
| 1. Woman dancing | | End | On the left | Blonde/ Green top and jeans… |
| 1. Landowski shopping center | | End | On the left | Written in upper case / green space in front of it /two trees / glazed building … |

**B. Snapshot of the events encountered in the virtual environment**

**
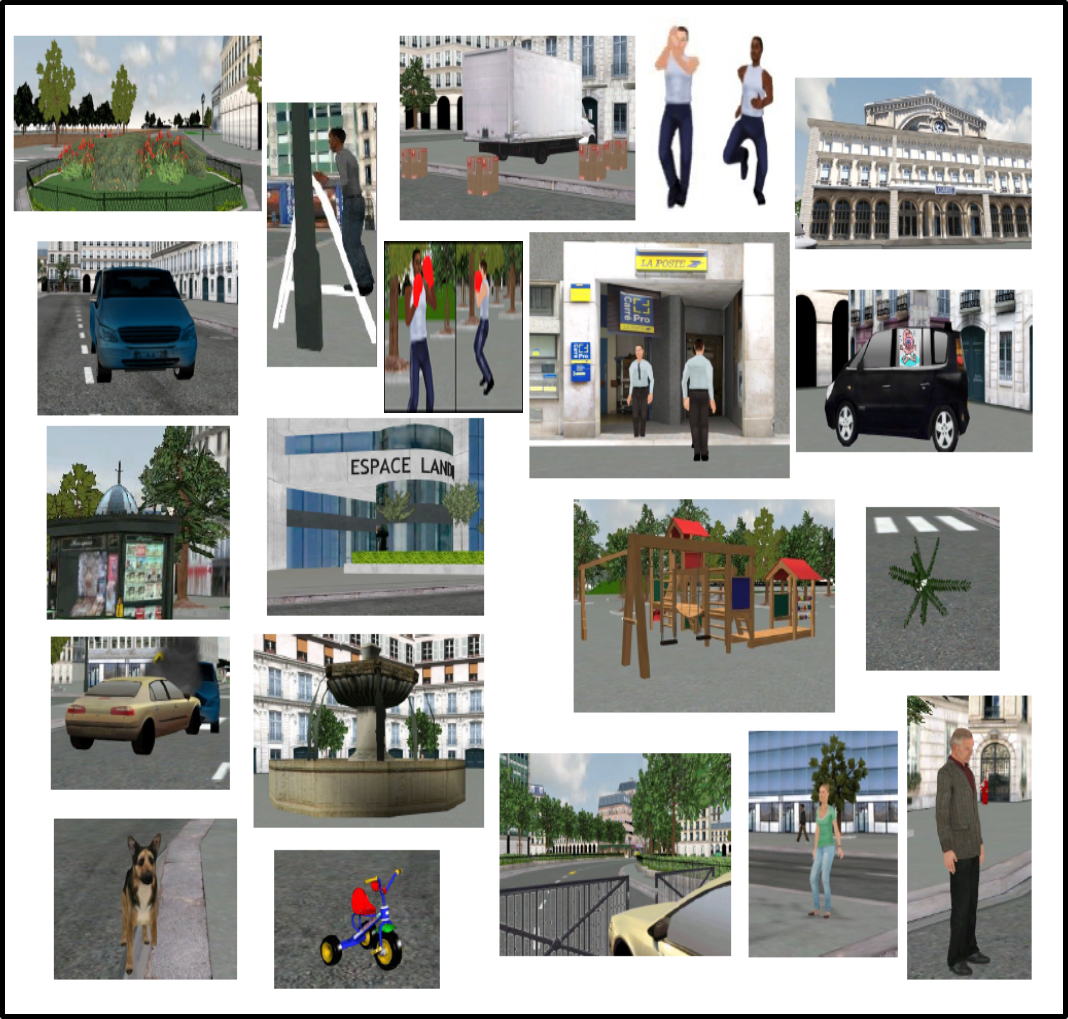
**

1. **Examples of transcripts**

**Extract of free recall by a younger adult**

*“****At the beginning of the route*** *I remember having seen a* ***flower pot*** ***falling*** *in* ***front of*** *me, it contains a* ***green plant*** *I think in a* ***brown vase****. When falling, it makes a* ***breaking*** ***noise****.* ***Just after*** *I remember having seen a* ***blue car*** *that comes* ***toward me****, it intrigued me because it was as if it was going to* ***hit me*** *, it was just* ***in front of me*** *[ ...]* ***Halfway through*** *my navigation, I remember that* ***there were two boxers on my right they were wearing red gloves*** *[…]* ***At the end of the route*** *I remember there was* ***a brown dog*** *that appeared* ***in front of me****, it* ***barked loudly*** *and was* ***without its master*** *[…]*

| *What* | *What-When* | *What-Where* | *What-details* | *Binding (What-Where-When)* | *High Binding (What-Where-When and Details)* |
| --- | --- | --- | --- | --- | --- |
| *Flower pot falling* | ***At the beginning*** | ***Ahead*** | *Green plant /brown vase Breaking noise* |  |  |
| *1* | ***1*** | ***1*** | ***1*** | ***1*** | ***1*** |
| *A car* | ***Just after*** | ***Ahead*** | ***Blue /coming toward me/going to hit me*** |  |  |
| *1* | ***1*** | ***1*** | ***1*** | ***1*** | ***1*** |
| *Boxers* | ***Halfway through*** | ***Right*** | *Wearing red gloves …* |  |  |
| *1* | ***1*** | ***1*** | ***1*** | ***1*** | ***1*** |
| *Dog* | ***At the end*** | ***Ahead*** | *Brown dog, barked loudly/without a master* |  |  |
| *1* | ***1*** | ***1*** | ***1*** | ***1*** | ***1*** |

**Scoring:**

**Extract of free recall by an older adult**

*“****Firs****t, I saw a* ***flower pot*** ***falling*** *in* ***front of*** *me, it made a* ***breaking noise****, I continued to walk then to* ***my left*** *I remember having seen a kind of* ***removal van*** *with* ***cardboard boxes*** ***down on the street****[ ...] Now, we are* ***halfway through*** *my navigation, I remember that* ***on my right*** *there was a* ***train station****, I think there were many columns like at the ‘Gard de LYON’ […]toward the end, there was a* ***boxer*** *also on* ***my right*** *he* ***had red gloves, he was moving or fighting****, there was* ***another one not far from him but not fighting with him****, I didn’t understand* ***what he was doing exactly*** *[...] towards the* ***end*** *I remember seeing* ***a woman I don’t remember what she looked like, it was*** *on* ***my left****, however, I* ***do not remember what she was doing*** *[....]”*

**Scoring:**

| *What* | *What-When* | *What-Where* | *What-details* | *Binding (What-Where-When)* | *High Binding (What-Where-When and Details)* |
| --- | --- | --- | --- | --- | --- |
| *Flower pot falling* | ***First*** | ***Ahead*** | *Breaking noise* |  |  |
| *1* | ***1*** | ***1*** | ***1*** | ***1*** | ***1*** |
| *Removal van* | ***First … I continued*** | ***Left*** | *cardboard boxes on the street* |  |  |
| *1* | ***1*** | ***0*** | ***1*** | ***0*** | ***0*** |
| *Train station* | ***Halfway through*** | ***Right*** | ***Many columns like the “Gare de Lyon”*** |  |  |
| *1* | ***1*** | ***1*** | ***1*** | ***1*** | ***1*** |
| *Boxers* | ***End*** | ***Right*** | *, fighting, red gloves …* |  |  |
| *1* | ***0*** | ***1*** | ***1*** | ***0*** | ***0*** |
| *Women* | ***Toward the end*** | ***left*** | *I don’t’ remember what she was doing nor what she looked like* |  |  |
| *1* | ***1*** | ***1*** | ***0*** | ***1*** | ***0*** |
